# Geographical differences in whooping cough in Catalonia, Spain, from 1990 to 2010

**DOI:** 10.1186/1471-2458-14-268

**Published:** 2014-03-20

**Authors:** Inma Crespo, Núria Soldevila, Pilar Muñoz, Pere Godoy, Gloria Carmona, Angela Domínguez

**Affiliations:** 1 CIBER de Epidemiología y Salud Pública (CIBERESP), Casanova 143, 3rd floor. Public Health Department, 08036 Barcelona, Spain; 2 Department of Public Health, University of Barcelona, Casanova 143, 08036 Barcelona, Spain; 3 Department of Statistics and Op. Research, Building C5 (North Campus). Jordi Girona 1-3, 08034 Barcelona, Spain; 4 Subdirectorate of Public Health, Department of Health, Generalitat of Catalonia, Roc Boronat 81-95, 08005 Barcelona, Spain

**Keywords:** Whooping cough, Surveillance, Epidemiology, Public health

## Abstract

**Background:**

Whooping cough is a communicable disease whose incidence has increased in recent years in some countries with vaccination. Since 1981, in Catalonia (Spain), cases must be reported to the Public Health Department. In 1997, surveillance changed from aggregated counts to individual report and the surveillance system was improved after 2002. Catalan public health is universal with equal coverage geographically. The aim of this study was to determine whether there are differences in whooping cough incidence in rural and urban counties.

**Methods:**

Cases in 1990–2010 were classified as rural or urban. Incidences and risk ratios (RR) between urban and rural counties and 95% CI were calculated. Associations between rural and urban counties and structural changes during the study period were analysed.

**Results:**

Twelve years of the whole study period showed differences in incidence between rural and urban counties. The incidence was higher in urban counties in seven years and rural counties in five years. There was a positive association of whooping cough incidence in rural and urban counties in four-week periods. Structural changes were detected in the following four-week periods: 4^th^ in 1993, 7^th^ in 1996 and 3^rd^ 2005 in rural counties and 5^th^ 1993, 9^th^ in 1996 and 8^th^ in 2007 in urban counties.

**Conclusions:**

Differences in whooping cough between rural and urban counties were found. In most years, the incidence was higher in urban than in rural counties. Rural and urban counties show similar cyclic behaviour when four-week periods were considered.

## Background

Whooping cough is a respiratory tract disease caused by *Bordetella pertussis,* a gram-negative bacterium, and was not preventable until the introduction of the whole-cell vaccine (DTwP).

Whooping cough presented epidemic peaks each two to five years in the prevaccination era. Introduction of the vaccine reduced the incidence but did not change these intervals, suggesting endemic circulation of *Bordetella pertussis*[1-4].

In Catalonia (Spain), DTwP vaccination was introduced into the vaccination schedule in 1965 [5-7], with vaccination at 3, 5, 7 and 18 months of age. From 1998 onwards, the vaccine was administered at 2, 4 and 6 months of age. In the same year, the acellular vaccine (DTaP) appeared and began to be administered to infants at 18 months, with a new booster dose at 4–6 years of age. In 2000, the DTaP and DTwP vaccines were administered without differentiation in infants younger than 1 year. This situation triggered the total replacement of DTwP by DTaP in the vaccination schedule in 2002 [6], because the DTwP vaccine was more reactogenic than the DTaP vaccine [1,8]. However, in the last decade, some studies have reported an increase in whooping cough incidence in spite of high vaccination coverages, especially in adults [9-15].

In Catalonia, cases of whooping cough must be reported to the Department of Health since 1981. The surveillance system has undergone changes over time to improve data quality and disease control. Reporting began in 1981 as aggregated counts. Until 1997, physicians had to report the weekly number of suspected or confirmed cases of whooping cough in patients they attended. From 1997 onwards, whooping cough reporting was individualized and made mandatory, and physicians had to make a specific report on each case [16]. In January 2003 [17], changes were introduced to increase case detection, facilitating diagnosis by PCR techniques and incrementing surveillance efforts by physicians to identify and report whooping cough [13].

The aim of this study was to evaluate differences in the incidence of whooping cough between rural and urban areas in Catalonia between 1990 and 2010.

## Methods

### Surveillance

The study was carried out between 1990 and 2010 in Catalonia, a region in North-eastern Spain with 7.5 million inhabitants. The case definition of whooping cough (available since 1997) was coughing for ≥2 weeks accompanied by ≥1 of the following symptoms: paroxysmal cough, inspiratory whoop, posttussive vomiting or apnoea [18]. As a mandatory disease, physicians must report cases detected. General data is published by the Public Health Department in the Epidemiological Bulletin of Catalonia, which is freely available. We collected the number of cases and the county of residence of each case between 1990 and 2010 from the Epidemiological Bulletin of Catalonia.

### Analysis

The reported cases of whooping cough in the surveillance period were aggregated into 13 four-week periods for each year.

The population was estimated using figures from the Statistical Institute of Catalonia (Idescat) [19]. Catalonia is composed of 41 counties. Counties were classified as rural or urban. Rural counties were those where the population density was < 100 inhab/km^2^ and the population was < 30,000 inhabitants [20].

Structural changes (SC) in rural and urban time series were detected using the breakpoints function of the strucchange package, in R v2.9.1 software. This function was used to identify trend changes over the study period; SC show specific four-week periods when the incidence of whooping cough changed its previous behaviour (increases or decreases) [21].

To determine the seasonality of the disease, several models were designed using aggregation of different numbers of four-week periods in rural and urban counties separately and together.

After testing different models, we used negative binomial regression to adjust rural and urban cases.

These models were constructed with the R statistical software, using the glm.nb function of the MASS package to adjust rural and urban cases as a generalized linear model (GLM) with the logarithmic link and the error adjusted by negative binomial distribution. Model selection and validation took into account the statistical significance of the covariates and the minimum Akaike information criterion (AIC).

Adjusted models:

Rural cases adjusted for urban cases, year of report, two sinusoidal variables to adjust for the cycling component, the population as an offset parameter and ε was an error term.

$$\begin{array}{ll} \;\log\left(\text{rural}\_\text{cases}\right) & =\beta_{0}+\beta_{1}\cdot \log\left(\text{urban}\_\text{cases}\right)\\ & \;+\beta_{2}\cdot \text{factor}\left(\text{year}\right)+\beta_{3}\cdot \text{sin}\left(\frac{2\pi t}{\text{amplitude}}\right)\\ & \;+\beta_{4}\cdot \cos\left(\frac{2\pi t}{\text{amplitude}}\right)+\varepsilon \end{array}$$

Urban cases adjusted for rural cases, year of report, two sinusoidal variables to adjust for the cycling component, the population as an offset parameter and ε was an error term.

$$\begin{array}{ll} \;\log\left(\text{urban}\_\text{cases}\right) & =\beta_{0}+\beta_{1}\cdot \log\left(\text{rural}\_\text{cases}\right)+\beta_{2}\cdot \text{factor}\left(\text{year}\right)\\ & \;+\beta_{3}\cdot \sin\left(\frac{2\pi t}{\text{amplitude}}\right)+\beta_{4}\cdot \cos\left(\frac{2\pi t}{\text{amplitude}}\right)+\varepsilon \end{array}$$

Incidence rates and their 95% confidence intervals (CI) were calculated for each year. The risk ratios (RR) and their 95% CI of incidences rates in rural and urban counties were calculated. Statistical significance was established as p < 0.05. Data were analysed using the SPSS v.18, Epidata and R programmes.

## Results

### Rural and urban incidences by year

**Figure 1:**
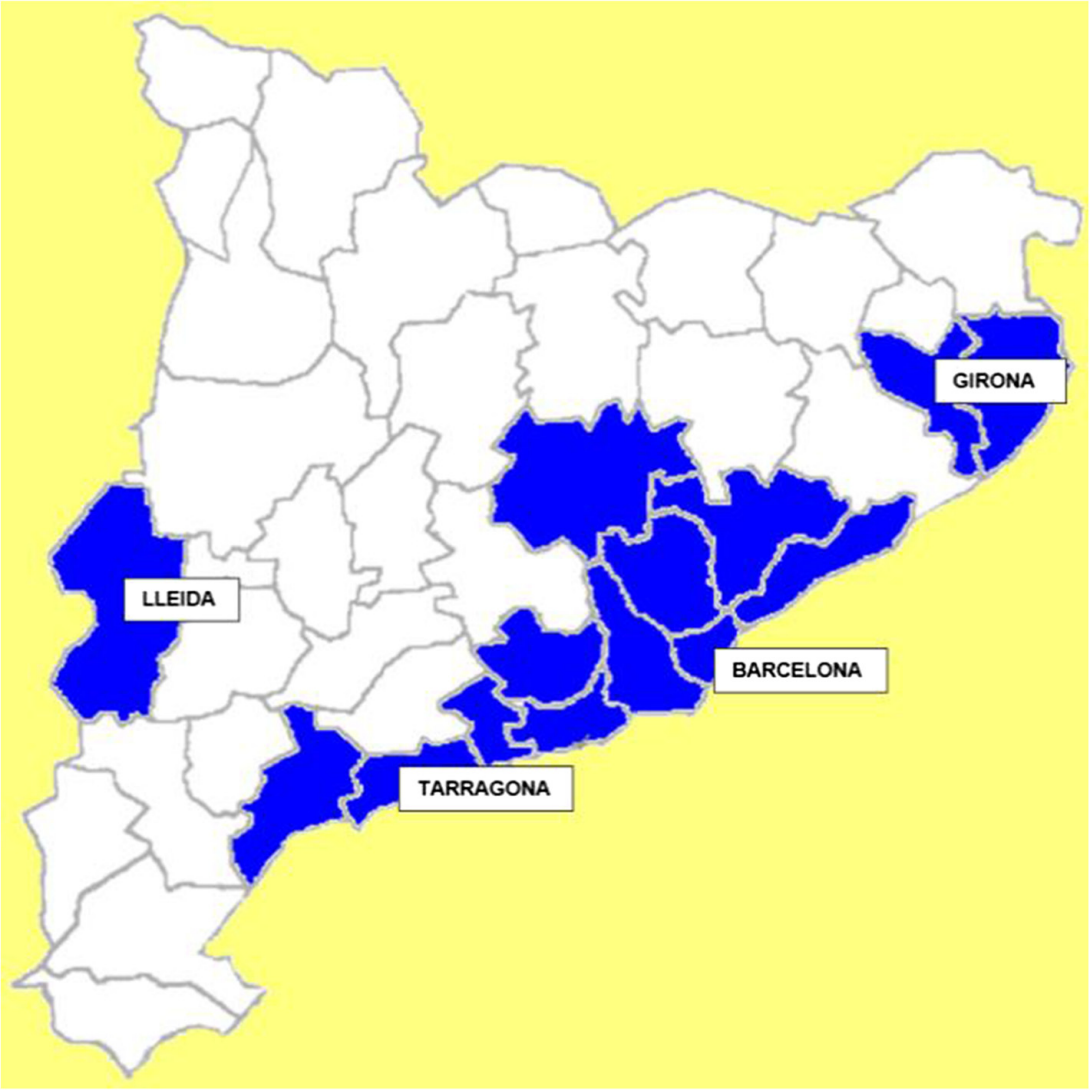
**Map of Catalonia.** Rural counties are in white and urban counties in blue. Provincial capitals are shown. Urban counties were aggregated near the larger cities.

**Figure 2:**
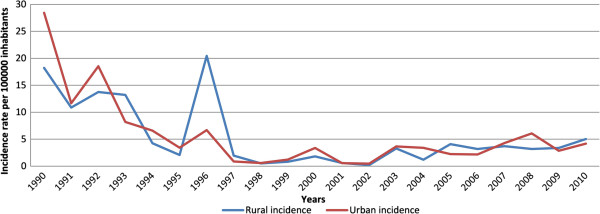
**Incidence of whooping cough in Catalonia by rural and urban counties and year.** Incidence rate per year reported to the surveillance sistem in Catalonia. Rural incidence are in blue and urban incidence in red.

Between 1990 and 2010, 7540 cases of whooping cough were reported in Catalonia distributed in 27 rural counties and 14 urban counties (Figure 1). Figure 2 shows the incidence rate of whooping cough in a time series from 1990 to 2010 in rural and urban counties. The incidence rate showed decreases and increases in parallel in rural and urban counties from 1991 to 2004. After this year, rural and urban cases did not show the same behaviour.

Incidence rates according to rural or urban counties are shown in detail in Table 1, which also shows the RR, the 95% CI and the p value, year by year. There were differences in the incidence in 12 years of the study period. The incidence rate was higher in rural counties in five years (1993, 1996, 1997, 2005 and 2006) and in urban counties in seven years (1900, 1992, 1994, 1995, 2000, 2004 and 2008). In the other 9 years (1991, 1998, 1999, 2001, 2002, 2003, 2007, 2009 and 2010) no differences were found.

**Table 1:** Annual incidence of whooping cough in urban and rural counties by year

**Year**	**Rural counties**	**Urban counties**	**RR (95% CI)**	**p value**
**1990**	18.21	28.43	0.64 (0.54-0.75)	<0.001
**1991**	10.86	11.67	0.93 (0.75-1.14)	0.50
**1992**	13.74	18.51	0.74 (0.61-0.89)	0.001
**1993**	13.21	8.18	1.61 (1.32-1.97)	<0.001
**1994**	4.25	6.59	0.64 (0.46-0.89)	0.007
**1995**	2.07	3.41	0.60 (0.38-0.96)	0.033
**1996**	20.44	6.69	3.05 (2.56-3.64)	<0.001
**1997**	1.95	0.86	2.24 (1.31-3.83)	0.002
**1998**	0.51	0.57	0.88 (0.34-2.28)	0.801
**1999**	0.81	1.22	0.66 (0.31-1.38)	0.261
**2000**	1.80	3.36	0.53 (0.33-0.87)	0.010
**2001**	0.59	0.56	1.05 (0.43-2.53)	0.901
**2002**	0.19	0.45	0.42 (0.09-1.77)	0.291*
**2003**	3.27	3.65	0.89 (0.62-1.28)	0.552
**2004**	1.19	3.39	0.35 (0.20-0.61)	<0.001
**2005**	4.06	2.23	1.82 (1.30-2.54)	<0.001
**2006**	3.18	2.17	1.46 (1.01-2.11)	0.039
**2007**	3.70	4.28	0.86 (0.62-1.18)	0.371
**2008**	3.18	6.07	0.52 (0.37-0.72)	<0.001
**2009**	3.36	2.84	1.18 (0.84-1.65)	0.321
**2010**	5.02	4.18	1.19 (0.91-1.58)	0.193

*Fisher test.

### Rural and urban counties analyses by four-week periods

Table 2 shows the results of adjusted models of cases distributed by four-week periods in rural and urban counties with a positive association of incidences in rural and urban counties.

**Table 2:** Adjusted models in rural and urban counties by four-week periods

**Rural counties model**	**Estimate**	**P value**
Intercept	-12.13	<0.01
Log (urban cases)	0.01	<0.01
Sinus	-0.24	<0.01
Cosines	-0.27	<0.01
Factor (year)		<0.01
AIC:	1211.2	
Null deviance: 592.88 on 272 degrees of freedom		
Residual deviance: 285.48 on 249 degrees of freedom		
**Urban counties model**	**Estimate**	**P value**
Intercept	-9.29	<0.01
Log (rural cases)	0.02	<0.01
Sinus	-0.25	<0.01
Cosines	-0.29	<0.01
Factor (year)		<0.01
AIC:	1972.2	
Null deviance: 1016.20 on 272 degrees of freedom		
Residual deviance: 306.77 on 249 degrees of freedom		

**Figure 3:**
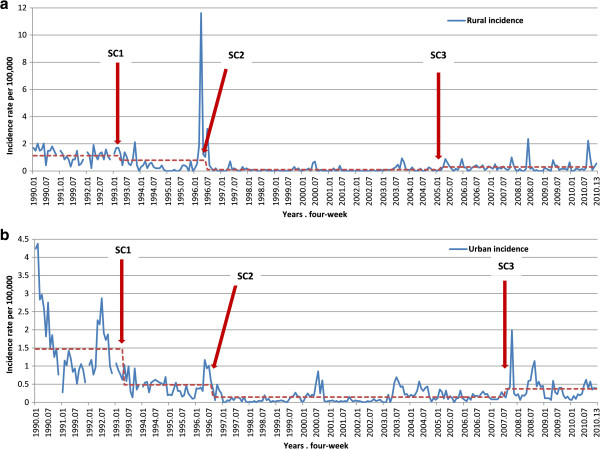
**Structural changes (SC) in the four-week periods in urban and rural counties of Catalonia, 1990–2010.** Each year was distributed in 13 four-week periods. **a**. Rural counties. SC occurred in the following four-week periods: 4th in 1993, 7th in 1996 and 3rd in 2005. **b**. Urban counties. SC occurred in the following four-week periods: 5th in 1993, 9th in 1996 and 8th in 2007.

Figure 3 show SC by four-week periods in rural and urban counties. In rural counties, structural changes occurred in the following epidemiological four-week periods: 4^th^ in 1993, 7^th^ in 1996 and 3^rd^ in 2005. In urban areas, SC occurred in the following four-week periods: 5^th^ in 1993, 9^th^ in 1996 and 8^th^ in 2007. Seasonality shows that the disease was more frequent in summer four-week periods (from 6^th^ to 9^th^) but this was not statically significant (p = 0.85 in rural areas, p = 0.33 in urban areas and p = 0.38 in both areas together).

## Discussion

During the study period, the incidence alternated between rural and urban counties.

Rural counties were further from Barcelona and other large cities than urban counties. The birth rate was higher in urban counties and therefore the number of susceptible people increased more rapidly than in rural counties, and the transmission of whooping cough was also higher and recurrent. Some studies have reported this situation with whooping cough and other infectious diseases [6,7,22-24]. Other studies have found that whooping cough is more frequent in rural than in urban areas [10,25] or have found higher whooping cough mortality rates in rural than in metropolitan areas [26]. However, mortality rates may not be a good indicator of disease incidence, which is influenced by additional factors as quality of health services, previous health status or others.

Our results showed differences in the incidence between rural and urban counties in most of the years studied. The incidence was high in urban areas in most years, coinciding with epidemic peaks of the disease when the bacterium was widely spread. In 2005 and 2006, the incidence was higher in rural counties, which may have been due to the outbreaks that occurred in 2005 [27]. The usual distribution of outbreaks was 20% in rural counties and 80% in urban counties but, in 2005, 53% of outbreaks occurred in rural counties. In 1993, 1996 and 1997, we assume the same occurred, but we cannot confirm this because outbreaks reports have only been available since 1997 [28]. The years in which no differences in the incidence were found coincide with the low incidence of the epidemic cycles and restricted community circulation of the bacterium. An exception was 2003, when there were no differences between rural and urban counties and the incidence was high in both. The change introduced in the surveillance system in that year may explain the better detection and reporting of the disease in both rural and urban counties [17]. A USA study suggests, as do our data, that improved surveillance and diagnostic facilities may result in an increase in the reported incidence of whooping cough [29].

With respect to the four-week periods, three SC were detected in rural and three in urban counties. The first SC was in the 4^th^ and 5^th^ in 1993, respectively, the second was in the 7^th^ and 9^th^ in 1993, respectively and the third was in the 3^rd^ in 2005 in rural and 8^th^ in 2007 in urban counties. The first SC observed in 1993 in both rural and urban counties might be explained by the ending of an epidemic cycle. The second SC in 1996, also in both, may show the changes introduced in epidemiological surveillance in Catalonia. The change in the surveillance system from aggregated counts to individual report probably had a direct impact on the number of reported cases, as observed in other preventable diseases in Catalonia, such as rubella and measles [16]. These data confirm the importance of considering operational changes in the surveillance system in interpreting incidences rates, as reported by American and European studies [29-31]. The third SC occurred in early 2005 in rural counties and in late 2007 in urban counties and may be explained by the outbreaks that occurred in each of these years. In 2005 rural counties had their maximum number of outbreaks (7) which was twice as many as the previous year. In urban counties, the number of outbreaks increased to 35 in 2007, which was two and a half times greater than the previous year [27,32].

In both, rural and urban counties, incidence increases or decreases at the same four-week period. A possible reason for the association found in the model adjusted by four-week periods may be a synchronised cyclic behaviour of whooping cough [1-4].

A possible explanation of differences in incidence in rural and urban areas may be the vaccination coverage. A Canadian study found a higher incidence of whooping cough in rural than in urban areas, but suggested this was due to large differences in vaccination coverage, with higher coverage in urban areas [10].

A 2003 study in Catalonia did not find differences in vaccination coverage in rural and urban settings. Coverage of the third dose of the DTP vaccine and a booster dose was 98.5% and 94.1%, respectively, in rural counties and 98.8% and 94.7%, respectively, in urban counties, but the differences were not statically significant [33].

Physicians in rural and urban counties have, theoretically, the same capacity to collect and send samples directly to the laboratory using personal messenger services, at least since 2003 when the surveillance system was improved in Catalonia [18,34].

Therefore, it seems unlikely that the differences found between incidences in rural and urban counties can be explained by differences in the vaccination coverage or the surveillance system.

## Conclusion

We found differences in the incidence of whooping cough between rural and urban counties in 12 of the 21 years studied. The incidence was higher in urban areas in most cases when there were differences. The cyclic behaviour in rural and urban counties was similar when four-week periods were considered. Improvements in the surveillance system would help to improve the follow up of the incidence of this vaccine-preventable disease and make appropriate recommendations for disease control.

## Abbreviations

RR: Risk ratio; CI: Confidence interval; DTwP: Diphtheria-tetanus-whole cell pertussis; DTaP: Diphtheria-tetanus-acellular pertussis; PCR: Polymerase chain reaction; SC: Structural change; AIC: Akaike information criterion.

## Competing interests

The authors declare that they have no competing interests.

## Authors’ contributions

IC contributed to the study design, performed the statistical study and manuscript writing. NS and PM contributed to the interpretation of the data analysis. PG, GC and AD contributed to the interpretation of the study results and critical revision of the manuscript. All authors have seen and approved the final version of the manuscript.

## Pre-publication history

The pre-publication history for this paper can be accessed here:

http://www.biomedcentral.com/1471-2458/14/268/prepub
